# Age Differences and Changes in Resources Essential to Aging Well: A Comparison of Sexagenarians, Octogenarians, and Centenarians

**DOI:** 10.1155/2011/357896

**Published:** 2011-11-13

**Authors:** G. Kevin Randall, Peter Martin, Alex J. Bishop, Leonard W. Poon, Mary Ann Johnson

**Affiliations:** ^1^Bradley University, C. C. Wheeler Institute, 05 Bradley Hall, 1501 W. Bradley Avenue, Peoria, IL 61625, USA; ^2^Gerontology Program, Iowa State University, 1096 LeBaron Hall, Ames, IA 50011-1120, USA; ^3^Department of Human Development & Family Science, Oklahoma State University, 328A HES, Stillwater, OK 74078, USA; ^4^Institute of Gerontology, College of Public Health, 255 E. Hancock Avenue, Athens, GA 30602-5775, USA; ^5^Department of Foods & Nutrition, Institute of Gerontology, 143 Barrow Hall, 115 DW Brooks Drive, Athens, GA 30602, USA

## Abstract

This study examined change over time in five resources assessed by the Duke OARS Multidisciplinary Functional Assessment Questionnaire: social, economic, mental, physical, and functional resources. Two hundred and one participants in the Georgia Centenarian Study provided data for this longitudinal study: 70 sexagenarians, 63 octogenarians, and 68 centenarians. Those in their 60s and 80s were followed up within 60 months; due to mortality attrition, centenarians were followed up within 20 months. Centenarians experienced the lowest levels of resources relative to those in their 80s and 60s. Over time they primarily experienced loss in activities of daily living, highlighting that the ability to maximize gains and mitigate losses over time for older adults is highly associated with various resources essential to well-being. Findings suggest that older adults'—especially the very old—resources should be concurrently assessed in a multidimensional analysis by researchers and practitioners who work with older adults in various settings.

## 1. Introduction

 Multiple, coexisting physical and psychiatric diseases are prevalent among “expert survivors” or centenarians [1, 2]. Yet, not all older adults change in similar fashion. Andersen-Ranberg et al. [3] maintained that “although healthy centenarians do not exist, autonomous ones do” (page 906). In keeping with such heterogeneity in the well-being of older adults, calls for renewed attention to multidimensional assessments of resources essential for aging well have surfaced in the literature. Especially critical is the fact that differences may exist in adaptation to aging among the young-old, old-old, and oldest-old. Thus, this study examined change over time in key resources essential to positive adaptation with age—social, economic, mental, physical, and functional resources—among older adults in their 60s, 80s, and 100s.

Krach and colleagues [4] examined six domains of functioning in 50 adults over the age of 85: physical, mental, social, spiritual, economic resources, and activities of daily living and concluded that “… multidimensional assessment is necessary to identify nursing interventions that will regain, maintain or enhance functioning among oldest-old people” (page 456). Also, Blazer [5] wrote “I propose that psychiatrists who treat the oldest old rejoin our colleagues in geriatric medicine by emphasizing health-related quality of life (specifically functional status) and a comprehensive, interdisciplinary approach to assessment and management of psychiatric disorders” (page 1915). He summarized the work by Patrick and Erickson [6] and defined health-related quality of life by core concepts that included the five multidimensional resources assessed by the Multidisciplinary Functional Assessment Questionnaire (MFAQ; [7])—a key tool developed by the Older Americans Resources and Services Program at Duke University (OARS; [8]). He recommended [5] comprehensive assessment, particularly in the case of those presenting with moderate to severe depressive symptoms, listing the OARS MFAQ (hereafter referred to as OMFAQ) as one of a few standardized scales focusing on the self-reported, health-related quality of life (page 1920). 

To date, no studies have been found that systematically investigate change over time in these comprehensive resources (i.e., social, economic, mental, physical, and functional) assessed by the OMFAQ among older adults—especially centenarians. The focus of this study was on the mechanisms or resources that older individuals draw upon to adapt to the normative decrepitude of advanced age. In particular, because aging adults typically struggle with increased hearing, vision, and functional health losses, these five resources are especially salient for positive adaptation to age-related losses.

Baltes [9] alerted researchers to the special constraints that challenge life for individuals, especially those in the fourth age or the oldest-old (i.e., 80 years of age and beyond). He offered three foundational and constraining principles for individual human development and adaptation to age-associated losses in extreme old age. First, the associated benefits from evolutionary selection decrease with age. Substantiating this claim with examples of age-associated loss, Baltes summarized his point: “Evolution and biology are not good friends of old age” (page 368). Conversely, as evolutionary benefits of natural selection decrease with age, Baltes' second principle stated that an individual's need or demand for culture increases. Baltes [9] defined culture as “… the entirety of psychological social, material, and symbolic (knowledge-based) resources …” (page 368). He explained that these resources were necessary for individual human development beyond adulthood and into old age. Juxtaposing these two principles, Baltes painted the picture of an aging individual who experienced losses associated with the depreciating benefits of inherited biological assets. However, concurrently, the same individual experienced an increasing need for culture-based compensations in the form of resources (e.g., material, technical, social, economic, and psychological) in order to maintain adequate and stable levels of functioning. The third constraining principle of lifespan development is somewhat of a composite of the first two. Because evolutionary benefits decrease but an individual's need for culture increases, the efficacy of culture (e.g., psychological, social, and material resources) also decreases with advancing age. According to Baltes [9], “multicausality, multidimensionality, multidirectionality, and multifunctionality reign supreme in ontogenesis at all stages of the life course” (page 368) and, in particular, in advanced age or among those experiencing the impact of all three constraining principles of the lifespan architecture—the oldest-old or centenarians. Based on the three developmental assumptions proposed by Baltes, we propose that the impact of all three constraining principles would be most relevant for centenarians.

Building upon this foundational concept of the incomplete architecture of lifespan human development, M. M. Baltes [10] and Hobfoll [11] extended the literature by examining the role of psychosocial resources and well-being. Baltes [10] summarized work regarding knowledge about age-associated decline and resources based upon studies done with those in their 60s and 70s and postulated about the role played by resources for those in the “fourth age.” She made the point that the ability to maximize gains and minimize losses is dependent upon the availability of resources stating “This is particularly so in the case of old age, because more and more resources will have to be enlisted to maintain the status quo.” (page 412). Further, she clarified the point that whereas age-related declines exist (and, likely, accelerate in advanced old age), resource-rich elders experience less of it. However, she cautioned that the positive aging experienced by those in their 60s and 70s may not be so easily achieved by those 85 years and older. Recently, social and psychological theorists have begun to address these questions concerning the use of resources by individuals to adapt to age-related declines as explicated by life-span psychologists. 

Hobfoll devised the conservation of resources theory [11] and provided a review of social and psychological resources and adaptation models and theories [12]. Resources are defined by him as “those objects, personal characteristics, conditions, or energies that are valued by the individual or that serve as a means for attainment of these objects, personal characteristics, conditions or energies,” [11, page 516], and he identified four in particular. Objects, like a home, for example, have value for what they provide and are intrinsically associated with economic resources. Personality and associated constructs such as locus of control comprise a second resource, personal characteristics, in Hobfoll's paradigm. Conditions subject individuals to certain roles relationally or culturally such as marriage, employment, or seniority and identify whether or not the condition ameliorates or exacerbates stress. Energies enable an individual to acquire other needed resources and include time, money, and knowledge. Last, he noted specifically that social resources do not necessarily fit into any one category but do serve as a resource that may either enhance or detract from other valued resources. It is within this resource and adaptation perspective that many researchers [13–16] have investigated the influential role that resources and age-related losses have on various measures of well-being.

Because the five resources (i.e., economic resources, mental health, physical health, activities of daily living, and social resources) assessed by the OMFAQ are so critical to the positive adaptation of adults advanced in years, we asked the question, how do the resources change over time for those in their 60s, 80s, and 100s? Also, how do these resources uniquely relate or contribute to change in each other over time? Although empirical studies have found that most resources are stable among older adults [17–19], previous frequency analyses of time 1 OMFAQ resources differed significantly across the three age groups (60s, 80s, and 100s); centenarians reported the lowest levels of resources [17]. Therefore, this study's first and second hypotheses predicted (a) a significant effect for age group on resources at time 1 and (b) a significant effect for age group on change in the OARS resources. In addition, this study posited that the centenarians would exhibit the most loss relative to the other two age groups. 

Numerous studies have used one or more measures of OMFAQ resources to investigate relationships between predictors and outcomes [20–22]. Also, these studies have primarily conducted cross-sectional analyses based upon theoretically driven conceptual models. However, to date, no study has been found that specified each of the five OMFAQ resources together in a robust analysis utilizing two time points in order to determine direction of association when controlling for the influence of each of the other resources. As a research question, this study specified such a model using latent growth curve analysis.

## 2. Method

The older adults who participated in the first Georgia Centenarian Study [23] were the focus of this study. Although the GCS participants provided information on a wide variety of measures, the focus of this study was a comprehensive assessment of the interrelationships of the multidimensional resources included in the OMFAQ [7]. In particular, mean differences in the resources between age groups, between age groups over time, and latent growth curve analyses were conducted using SPSS 19.0 and M*plus* 6.11 [24] to examine this study's research questions and to test hypotheses.

The University of Georgia's Survey Research Center, utilizing current voter registration rosters, obtained a representative sample of the younger age groups (i.e., those in their 60s and 80s). Potential participants were selected by random digit dialing, providing a representative sample of older adults in Georgia in terms of gender and race. Small groups of participants completed their questionnaires at community testing locations. Centenarians were selected through the assistance of the University of Georgia's Survey Research Center, the Office of the Governor of Georgia, the media, and local older adult service organizations [25, 26]. After the sampling frame was created, letters were sent to each potential participant describing the study. Next, arrangements to administer the Mini-Mental Status Examination (MMSE) [27] were made by telephone. Selection criteria for the final sample of community-dwelling individuals (at Time 1) included a score of 23 or higher on the MMSE or a score of 2 or lower on the Global Deterioration Scale [28]. Thus, community-dwelling and cognitively intact older adults who participated in the Georgia Centenarian Study [23, 29] were included in this study.

### 2.1. Measures

#### 2.1.1. Economic Resources

This measure included three dichotomous items tapping the sufficiency of the respondent's economic resources to meet emergencies and provide extras currently and in the future; two items scaled (1 = *poorly* to 3 = *very well*) asked the respondents how well they were doing financially and the last item scaled (1 = *cannot meet payments* to 3 = *payments are no problem*) assessed how heavy the participant's expenses were. This item tapped the respondent's ability to meet payments. These were summed to create the variable *Economic Resources*. For those in their 60s, Cronbach's alpha at time 1 =  .86 and at time 2 =  .78. For participants in their 80s, Cronbach's alpha at time 1 =  .81 and at time 2 =  .77. For participants in their 100s, Cronbach's alpha at time 1 =  .76 and at time 2 =  .80.

#### 2.1.2. Physical Health and Mental Health

A single-item measure of self-rated physical health, as a summary assessment of overall health status, has been shown to reliably predict outcomes such as mortality, BMI, physical activity, and hospitalization among others [30–32]. One study [33] compared the predictive accuracy of a single-item measure of general health with multi-item scales (e.g., mental component summary and physical component summary). The single-item performed as well as the multi-item measures regarding validity and reliability, in addition to saving time and money over the use of longer instruments. We used an item from the OMFAQ [7] physical health section asking “How would you rate your overall health at the present time?” Responses ranged from 0 (*poor*) to 3 (*excellent*). Similar to research done on a single-item of self-rated physical health, numerous studies have employed a one-item, global assessment of mental health [34, 35]. The current study measured self-reported mental health with an item from the OMFAQ [7] asking “How would you rate your mental or emotional health at the present time?” Responses ranged from 0 (*poor*) to 3 (*excellent*).

#### 2.1.3. Instrumental Activities of Daily Living (IADLs)

This measure summed seven items from the OMFAQ. Examples of items included (a) “Can you use the phone?” (b) “Can you get to places out of walking distance?”, and (c) “Can you go shopping for groceries or clothes?” The items were scaled (0 = *completely unable* to 2 = *without help*). For those in their 60s, Cronbach's alpha at time 1 =  .62 and at time 2 =  .65. For participants in their 80s, alpha at time 1 =  .78 and at time 2 =  .85. For participants in their 100s, alpha at time 1 =  .80 and at time 2 =  .87.

#### 2.1.4. Social Resources

Three questions from the OMFAQ interaction dimension of social support assessed social support structure—the size of support network and frequency of contact. One question asked about the number of past-week phone conversations, a second assessed the number of individuals the participant knew well enough to visit with in their homes, and a third question assessed the number of past-week visits participants with those whom they did not live. Each question was scaled from 0 to 3 with higher scores reflecting more contact. These items were summed to create an index of social resource structure. Work on this measure has found low internal consistency reliabilities, ranging from  .44 to  .61 [36]. Randall et al. [37] agreed with Burholt and colleagues [36] that these items were not designed to provide maximum internal consistency but rather provide a breadth of assessment and may not even be related. For example, phone conversations may be inversely related to personal visits as one may account for the other. With very old adults who tend to experience hearing-related losses, phone conversations may be limited relative to personal visits. In addition, we concur with Herbert and Cohen [38] who discuss the reasons for low internal consistency in checklists; we do believe these three items may best serve as a checklist of social resources and, thus, may not estimate a unidimensional construct, making expectation of typical internal consistency for such a three-item scale unwarranted.

#### 2.1.5. Age Group

Age group included three groups: participants in their 60s, participants in their 80s, and participants in their 100s. Age group was used as an exogenous predictor to detect associations of the resource variables with age.

### 2.2. Analyses

First, we ran descriptive statistics and zero-order correlations for all study variables. In order to test the study's first hypothesis regarding a significant effect for age group on resources at time 1, we conducted a factorial ANOVA for each resource. In addition, to test hypothesis two, we conducted a repeated measures ANOVA to examine whether or not an age group by time interaction occurred for each resource assessed. SPSS 19.0 was employed for these analyses. For between-group comparisons as a followup to the ANOVAs, we employed the post-hoc Scheffé procedure, conservatively controlling for Type I errors.

This study applied a latent growth curve framework to further investigate change and predictors of change among the five OMFAQ resources. Lorenz and colleagues [39] demonstrated that autoregressive models of change were sensitive to the magnitude of the stability coefficients and, in general, models specified as growth curves were more likely than autoregressive models to evidence significant paths between predictor variables and change in the outcome variable. Stoolmiller and Bank [40] also recommended that individual growth curve models are often a more useful alternative to studying change because they do not force the predictor variable to compete with the initial level of the outcome of interest. In fact, Stoolmiller and Bank asked this very challenging question, “How could X change with the mere passage of time? In order to have X change from time 1 to time 2, we need a causal agent that is logically distinct from X itself; otherwise we are supposing that X can change for no reason” (page 271). They also included Mulaik's [41] argument that philosophically, spontaneous change of an object is not plausible “A reason for the change must be something other than that which it explains” (page 271). 

Stoolmiller and Bank [40] pointed out that change measured across two time points and analyzed in a growth curve analysis is, essentially, an analysis of simple difference scores. Over the years, there have been a number of critics of difference scores, suggesting that they are unreliable and sensitive to regression toward the mean [42–44]. However, a number of authors have spoken out in defense of change scores, especially when researchers are limited to two repeated measures [45–47]. Because there is considerable support for change scores and because of the recent advances in analyzing change in a growth curve environment, this study specified and tested models of level or intercept and slope using repeated measures on two occasions in the growth curve framework.

Recent methodological advances in growth curve modeling [48, 49] allow developmentalists to examine interindividual differences in intraindividual change over the life course. As Krause [50] demonstrated, whereas mean differences may not exist over time, between-individual differences in within-individual change often exist and they may be profound. Thus, we first specified univariate growth curve models for each resource to test for significant variation in intraindividual change between individuals. Note that in order to estimate these models with two time points, the errors for the repeated measures are fixed to zero [48]. These just-identified models correspond to Rogosa's “improved-difference-score model” [40, 47]. As noted before, the intercept loadings were fixed at one; the loadings for the change factor were fixed to zero (time 1) and to one (time 2), and the errors were fixed to zero. The overall analysis then is similar to regular OLS regression where the manifest variables are assumed to be without measurement error and dissimilar to structural equation modeling where the error in the measured variables is modeled—a limitation of OLS regression and growth curve analyses with only two time points. 

However, a strength of the study is that these analyses employed M*plus* 6.11 [24] with full information maximum likelihood to account for missing data and the estimator MLR that calculates maximum likelihood parameter estimates with standard errors that are robust to nonnormality. Finally, because of strong evidence for significant interindividual differences in intraindividual change, we tested for significant predictors of growth factors of each OMFAQ resource by regressing the growth factors of each individual resource on the other OMFAQ resources at time 1. In sum, we conducted five separate analyses, one for each resource that regressed the growth factors (e.g., intercept and slope) of one resource on the time 1 measures of the other four resources and age group in order to see the unique association of these five predictors at time 1 on the growth factors of each resource.

## 3. Results

Community-dwelling and cognitively intact older adults included in this study were participants in the Georgia Centenarian Study [23, 29]. Community-dwelling (i.e., self-sufficient or partially self-sufficient, living in the community and not in custodial institutions) participants might live in their own homes, those of relatives, or other residential community settings. Cognitively intact individuals were defined as not demented or disoriented based on scores on the Mini-Mental Status Examination at the time of recruitment. The first wave of data collection included 321 older adults (217 women, 104 men), classified as sexagenarians (*n* = 91), octogenarians (*n* = 93), and centenarians (*n* = 137). At time 2, there were 70 sexagenarians, 63 octogenarians, and 68 centenarians. Almost one-third of the sample was Black (27.7% and 30.8% at time 1 and time 2, resp.). The majority of the sample was female (67.6%) at time 1, well educated (at least graduated from high school) and rated their health as excellent or good. A summary of demographic characteristics of the samples at time 1 and time 2 can be found in Table 1.

The younger two age groups were assessed again, five years later; the centenarians were assessed after approximately 20 months. The second wave involved 201 of the original participants (63% of the baseline sample). The differential time frame for assessment based on age group was part of the project's design, intended to allow time for expected change and to optimize sample size (remaining life expectancy for centenarians is much shorter relative to the younger age groups). An analysis was conducted to assess differences between individuals who only participated at time 1 and those who participated at time 2. Demographic characteristics, mental status, and the study variables at time 1 were used in a logistic regression to predict participation at time 2. Age group and mental health were significant predictors of participation in the second assessment, *B* = −.03, *P* = .003, for age group and *B* = .45, *P* = .03, for mental health. Physical health approached statistical significance, *B* = .35, *P* = .07. Thus, younger and mentally healthier individuals tended to participate at time 2. No other demographic or study measures were significant predictors of participation in the second assessment.

Descriptive statistics (range, means, standard deviations, skew, and kurtosis) for each resource are provided in Table 2. The variables appear to be univariate normal at both time 1, and time 2. Zero-order correlations between study variables at time 1 and time 2 are found in Table 3. It is noted that for both time 1 and time 2 the higher the age group, the less the resource as all correlations are negative, although some are not significant. Of note is that age group and IADLs are strongly and negatively associated (*r* = −.73, *P* ≤ .001 at time 1 and *r* = −.73, *P* ≤ .001 at time 2) as are age group and social resources (*r* = −.20, *P* ≤ .01 at time 1 and *r* = −.29, *P* ≤ .001 at time 2). A second observation is that the resources alone are intercorrelated positively, and mostly, significantly. 

Next, to test hypothesis 1, mean differences between age groups were examined for each resource dimension at time 1 by conducting a factorial ANOVA (see Table 4). With the exception of economic resources and mental health, significant mean differences were found between age groups for the OMFAQ resources. Post-hoc Scheffé analyses indicated that the centenarians had the lowest mean levels of IADLs, physical health, and social resources compared to the other age groups. Also, centenarians had lower levels of mental health relative to those in their 60s but not those in their 80s. Finally, age group explains a significant amount of the variance in IADLs (*η* ^2^ = .58); centenarians had a significantly lower score on IADLs than both those in their 80s and those in their 60s. 

In order to test hypothesis 2, we conducted a repeated measures ANOVA of the mean changes over time in the five resources by age groups; results are presented in Table 5. First, no effects were found for time or the interaction of time and age group on economic resources, mental health, and social resources. Second, a main effect of time on physical health was found (*P* < .05); physical health declined approximately 10% on average for those in their 60s, 2% for those in their 80s, and only 6% for the centenarians; although it was noted that at the first time point (see Table 3) centenarians scored significantly lower on physical health than both sexagenarians and octogenarians. Finally, a significant interaction of time and age group on IADLs was found (*P* < .001). Over time, the centenarians declined in IADLs approximately 43% on average compared to the relative stability of the sexagenarians and the octogenarians (9% reduction). This is especially noteworthy when considering the time between measurement occasions for the centenarians (i.e., approximately 20 months) compared to the younger age groups (five years). Results from post-hoc Scheffé tests revealed that those in their 60s scored significantly higher in IADLs compared to those in their 100s (*P* < .001) and those in their 80s scored significantly higher in IADLs compared to those in their 100s (*P* < .001).

These initial analyses of the OMFAQ resources clearly demonstrated the positive and often significant associations between the resources, and the differences between the age groups at time 1 and differences in change over time between the age groups. Not surprisingly, the centenarians experienced the lowest levels of resources relative to those in their 80s and 60s. However, relative to the other age groups, they did not experience significantly lower levels of resources over time (except for IADLs). It must be noted once again, that the assessment periods differed for those in their 100s (approximately 20 months apart) than those in their 60s and 80s (60 months apart). In light of the IADLs finding, the deleterious effect of time and age on functional health for centenarians is significant and substantive. As mentioned earlier, these analyses focus on average change over time. Thus, to further investigate change in the resources over time, we conducted an investigation of between-individual differences in intraindividual change.

First, we specified a univariate growth curve model for each construct. Each resource evidenced significant interindividual differences in intraindividual change as noted by the significant variance for both intercept and slope. Results for these analyses are found in Table 6. The association between intercept and slope was negative and significant for economic resources, physical health, mental health, and social resources. This means that on the average, initial levels of each resource was associated with a decline in that resource over time; in fact, the higher the initial level, the steeper the decline. However, such was not the case for IADLs; no association was found for initial level of IADLs and change in IADLs over time. Thus, regardless of initial level of IADLs, the average change was decline over time. Finally, because of strong evidence for significant interindividual differences in intraindividual change for each resource, we tested for significant predictors of the growth factors of each resource by regressing the growth factors (initial level or intercept and slope) of each individual resource on age group and the other OMFAQ resources at time 1.  Table 7 contains the results for the regressions of the four resources and age group on the growth factors of the fifth resource (i.e, this was done for each resource). Thus, similar to OLS regression, the results in Table 7 provide the estimates of the unique association of each predictor with the outcomes (growth factors of the resources) holding constant or controlling for the influence of the other predictors (i.e., the other four resources and age group).

**Figure 1:**
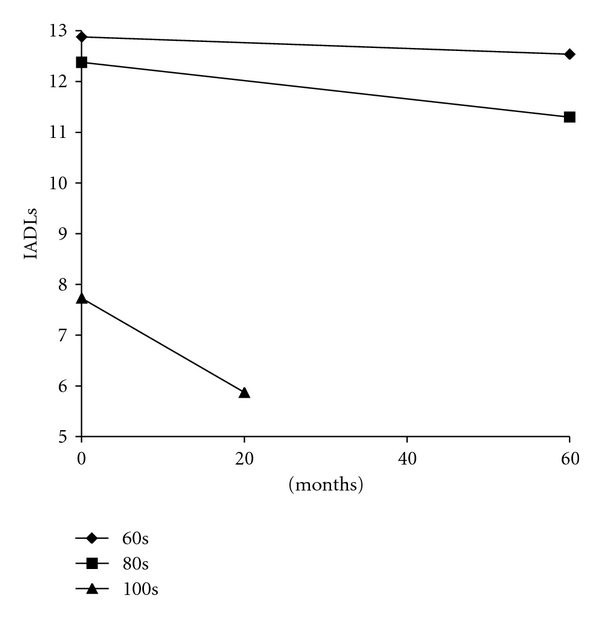
Time by age group interaction.

**Figure 2:**
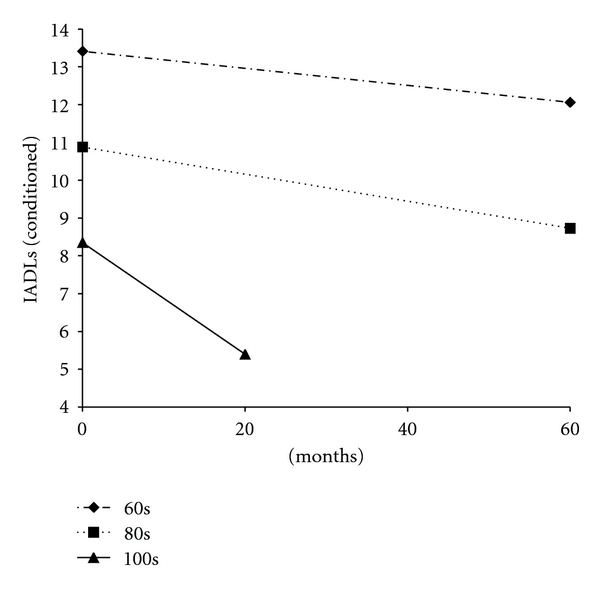
Age group predicts change in IADLs (controlling for all other resources).

Significant predictors of change (slope) were found for mental health, IADLs, and social resources. Age group at time 1 was negatively associated with slope for IADLs (*γ* = −.32; *t* = −4.26), mental health (*γ* = −.18; *t* = −1.67), and social resources (*γ* = −.20; *t* = −1.81). Levels of each resource decline over time, especially for the centenarians. In sum, because significant variation in both intercept and slope existed for each resource construct, we were able to investigate direction of influence and the interrelationships of the OMFAQ resources. The latent growth curve framework provided the opportunity to test, simultaneously, predictors of both level at time 1 and change in each resource. Age group was a main predictor of slope or change in IADLs, mental health, and social resources. The steepest declines in these outcomes over time were experienced by the oldest participants. In particular, the results supported the differential association between age group and change in IADLs (Figure 1) and the conditioned results of change in IADLs over time controlling for the influence of all other resources (Figure 2). Noteworthy is the comparison between the two figures. Figure 2 provides results from the growth curve analyses of the estimated conditioned means of IADLs by age group accounting for the influence of the other resources.

## 4. Discussion

The purpose of this study was to systematically investigate change in the five main resources older individuals rely upon for healthy adaptation to the normal declines experienced with age (i.e., social resources, economic resources, mental health, physical health, and activities of daily living). Also, the empirical focus of this study was on a widely used procedure for multidimensional functional assessment of older adults—the Multidisciplinary Functional Assessment Questionnaire [MFAQ; 7]—a key tool developed by the Older Americans Resources and Services Program at Duke University [OARS, 8; OMFAQ hereafter]. Numerous studies have employed one or more dimensions from the OMFAQ [20, 51–53]. However, to date, no studies were found that specifically examined change in the structural interrelationships of the five self-reported resources assessed by the OMFAQ among older adults—especially centenarians. As hypothesized, centenarians tended to score the lowest on resources at both time points and particularly demonstrated decline over time in IADLs, relative to those in their 60s and 80s. Specific results are now addressed.

Four specific results, based on the tests of mean differences in the OMFAQ dimensions between age groups at the first measurement occasion and over time, are noteworthy. First, no mean differences between the age groups were found for economic resources. This finding is consistent with a focused investigation of the economic well-being of the three age groups in this sample [54]. In their investigation of the OMFAQ economic resource self-assessed items at time 1 in the Georgia Centenarian Study, Goetting and colleagues [54] included the items used in this study's model. Overall, they only found age group differences on two out of 10 items assessed and one of those age group differences was based on only one response category. Finally, Goetting et al. [54] provided rationale for the lack of significant differences in economic resources among the three age groups. It could be that centenarians (who reported the lowest economic resources) employed a downward comparison to others who are worse off than themselves. Perhaps the centenarians made relative comparisons to other previous economically difficult periods in their lifespan (e.g., two World Wars and a Great Depression), mitigating concerns over their current economic situation. In addition, Tornstam's gerotranscendence perspective would suggest that very old adults turn away from effort to secure wealth and instead view financial resources as necessary for survival but nothing beyond that [55].

Second, in keeping with other studies of older age groups, including centenarians, and their levels of resources relative to younger age groups [17, 51], in this study's sample the mean levels of IADLs, physical health, and social resources were significantly lower for centenarians. In particular, centenarians in this study were extremely low in IADLs relative to those in their 60s and 80s. These results may further support the “compression of morbidity” hypothesis [56]. Rather than experience gradual declinations in overall health and gradual increases in disease-related mortality, individuals appear to experience a longer lifespan of good health truncating in a short period of poor health prior to death [56]. Future research on distal lifestyle practices and behaviors of centenarians is warranted, as these expert survivors may well have differentiated themselves from others in their birth cohort on certain conditions. This “compression of morbidity” likely explains the significant difference in IADLs between centenarians and both those in their 60s and 80s. Whereas there is not a significant difference between sexagenarians and octogenarians in IADLs, centenarians report, on average, 32% lower functional ability at time 1 relative to the younger age groups.

Third, the repeated measures analyses affirmed the results previously discussed regarding mean differences in IADLs at time 1. The significance test and the corresponding effect size for the interaction of age group and time represented, according to Cohen's [57] criteria, a large effect size. Age group differences explain most of the differences in IADLs over time. Post-hoc examinations revealed that once again centenarians were significantly and substantively lower on IADLs than those in either their 60s or 80s. Further support for the compression of morbidity explanation previously discussed was found in the less than significant difference in IADLs between those in their 60s and 80s. 

Recently, Fry and DeBats [20] investigated sources of life strengths as predictors of late-life mortality and, among other measures, employed the OMFAQ scale to assess self-rated disability (i.e., the IADL scale). In their sample of 380 randomly selected volunteers between the ages of 65 and 87 years, Fry and DeBats found a number of independent predictors of survival including disability (i.e., low levels of IADLs); low levels of these predictors created the greatest risk for male mortality. Finally, a longitudinal investigation predicting change in ADLs (in this particular study, IADLs and PADLs were combined) among the oldest old in Sweden also affirms the current study's findings that mean levels of IADLs change for the older participants relative to the younger age groups. Femia et al. [58] did find aggregate decline over time, but noted positively that the data included participants who either remained stable or improved in functional health over time. A significant predictor of stability in ADL functioning over a four-year time period was residential status, in particular community dwelling. In the current study's sample, relative stability is observed for the younger age groups (60s and 80s); all participants were community dwelling at the baseline assessment. However, a large effect was found for age; the centenarians experienced significant losses in IADLs compared to those in their 60s and 80s. Again, it must be noted that the difference in timing between measurements was five years for the sexagenarians and the octogenarians whereas approximately 20 months separated measurement occasions for the centenarians. Thus, significant change in IADLs occurred for the centenarians in this study.

Finally, consistent with previous investigations [17, 59] and the previously discussed “compression of morbidity” or accelerated decline of resources necessary for adapting to age-related changes at the very end of the lifespan, the centenarians reported the lowest levels of each of the other resources at time 1 (i.e., physical health, mental health, and social resources) relative to the younger age groups. However, only two resources significantly declined over time in this study's sample: physical health and IADLs. Although loss over time in physical health was experienced across the age groups, the centenarians reported the lowest levels of physical health at both time 1 and time 2. Second, as noted previously, centenarians reported significant losses in IADLs over time relative to the younger age groups. These results are similar to the findings of Steverink et al. [59] who assessed multiple resources relevant to the aging process and concluded “The higher the age, the more inclined the people were to frame the aging process in terms of physical decline and social loss and less in terms of continuous growth, regardless of most of their resources” (page P371). 

This study applied a latent growth curve framework to further investigate change and predictors of change in the OMFAQ resources. Heterogeneity within the sample for both intercept and slope in each of the OMFAQ resources, consistent with Krause [50], was demonstrated by the finding of significant interindividual differences in intraindividual change. Change in resources is complex among the age groups: some individuals are increasing in their levels of each resource, some are remaining relatively stable, and some are decreasing. Thus, this study proceeded to examine time 1 predictors of change in each resource. Three findings from these analyses are discussed: (a) economic resources predicted change in mental health, (b) physical health predicted change in mental health, and (c) age group was a primary predictor of change in mental health, IADLs, and social resources. 

In regard to the influence of age group, economic resources, and physical health on decreases in Mental Health over time, Steverink et al. [59] felt that the most important physical and material resources for older adults included health and financial security and that “Adapting to the process of aging is generally easier when a person is healthy and without financial worries” (page P365). However, at first glance, for this study's participants that maxim did not appear to hold. The higher the levels of these resources at time 1, the greater the decrease in mental health over time, controlling for the other resources. The strongest finding, in terms of effect size, was for economic resources. For participants in their 80s and 100s, fixed incomes likely contribute to this association as time erodes the buying power of the currency. In addition, obsolescence of material goods, particularly for community-dwelling individuals, also contributes to the lessoning of financial reserves. In similar fashion, over time, for each age group, physical health tended to decline, particularly for the centenarians. This finding is likely explained by the fact that for each age group, physical health significantly declines over time and is highly associated with mental health. 

Age group was a significant predictor of change in mental health, IADLs, and social resources. The relationship over time between age group with mental health and age group with IADLs is consistent with the previously discussed results; independence and autonomy as assessed by IADLs, as well as outlook on life assessed by mental health, are decreasing, especially for the centenarians. Relative to social resources, the significant association between age group and decline in slope of social resources was not surprising, especially when the influence of the other resources was controlled. Carstensen et al. [60] provided ample explication in their theory of socioemotional selectivity. Time is limited, particularly for those in their 80s and especially for those in their 100s, and the theory predicts change in social preferences and social network composition, focusing on only those relationships most salient to the older adults' well-being [61]. Persons surviving into advanced old age become more reliant on those social ties within their networks that provide the greatest emotional benefit. In turn, very old adults come to rely on a fewer number of close social affiliations, yet these relationships are considered essential to meeting one's goals near the end of life. 

This study affirmed its hypotheses with robust findings that provide implications for researchers and practitioners alike. Several limitations, however, exist that affect the generalization of this study's results. First, the participants were Southeastern older adults in reasonably good health, mentally competent, and community dwelling. Second, the younger age groups (those in their 60s and those in their 80s) were randomly selected by race and gender to approximate older adults in Georgia. However, in contrast, centenarians were selected using convenience sampling through state and local agencies. Also, the sexagenarians and octogenarians were assessed in testing locations; centenarians completed their assessments at home. In addition, for the two younger age groups, measurement occasions were five years apart but for the centenarians the measurement occasions were approximately 20 months apart. With only two waves of data, longitudinal results and age group comparisons are to be interpreted with caution. The functional form of change might not be linear, limiting the study's ability to test nonlinear or curvilinear models possible with multiple time points. 

The reduction in sample size, primarily due to mortality for the older age groups, from the first measurement occasion to the second (a 38% reduction from 321 to 201), opens the door for sample selectivity, also threatening the representativeness of the study's results [62]. In particular, Lindenberger and colleagues [63] specify that in longitudinal studies where mortality rates are greater than zero, selectivity results from two different sources: mortality-associated selectivity and experimental selectivity. Participants who have higher mortality risks may differ on variables of interest than participants with lower mortality risks, thus introducing a potential measurement confound; this type of selectivity is labeled mortality-associated. Experimental selectivity arises when subjects who participate in data collection differ on relevant variables of interest from subjects who are still living but are unable or unwilling to participate at later waves of data collection. In the case of the present study, it is highly probable that mortality-associated selectivity has influenced the findings. The result is that average levels of the multidimensional resources assessed are likely overestimated relative to the general population and that longitudinal change may be underestimated.

Finally, this study was not able to differentiate distinctly between age effects and the possible influence of cohort effects for any of the associations examined. For example, George [64] cited the conundrum of the fundamental influence of SES on illness vis-à-vis research that suggests differences in the association between SES and health across birth cohorts and summarizes her discussion by stating “Nonetheless, more effort is needed to understand cohort differences in the links between SES and health” (page 136). In order to disentangle the age and cohort confound, future research might consider a cohort sequential design that follows different cohorts across equal time intervals (e.g., follow a sample of adults who transitioned to young adulthood during the Great Depression, and other cohorts who transitioned to young adulthood ten and twenty years later).

Findings from this study produced several results strengthening the argument Baltes and Smith [65] and others [66–68] have proffered that aging well demands a multidimensional perspective and assessment, especially for the very old. The ability to maximize gains and mitigate losses over time for older adults is highly associated with various resources [11]. These resources should be concurrently assessed in a multidimensional analysis by researchers who study older adults and practitioners who work with them in various settings [4, 5]. This is especially important for those in the fourth age who experience ever-increasing demands on resources essential to well-being.

## Figures and Tables

**Table 1:** Sample demographic characteristics.

Variables	Time 1		Time 2		*χ* ^2^
	*n*	%	*n*	%	
Sex					1.59
Male	104	32.4	60	29.9	
Female	217	67.6	141	70.1	

Race					2.61
Black	89	27.7	62	30.8	
White	232	72.3	139	69.2	

Age Group					18.86***
60s	91	28.3	70	34.8	
80s	93	29.0	63	31.3	
100s	137	42.7	68	33.8	

Education					6.19
0–8 years	90	28.2	55	28.8	
High School	84	26.3	42	22.0	
Business/trade school	23	7.2	14	7.3	
College	75	23.6	44	23.0	
Graduate school	47	14.7	36	18.8	

Self-rated health					4.7
Excellent	67	21.1	39	20.2	
Good	159	50.2	93	48.2	
Fair	79	24.9	52	26.9	
Poor	12	3.8	9	4.7	

Because of rounding, percentages may not add to 100.

***P < .001.

**Table 2:** Descriptives for study variables study at time 1 and time 2.

Variables	*n*	Range	Mean	(SD)	Skew	Kurtosis
(1) Economic resources time 1	199	1–10	7.97	2.11	−1.27	1.02
(2) Economic resources time 2	194	2–8	7.74	2.20	−.99	−.02
(3) Mental health time 1	196	0–3	2.03	.72	−.47	.25
(4) Mental health time 2	191	0–3	1.97	.72	−.22	−.38
(5) IADLs time 1	199	1–13	10.96	2.99	−1.34	.61
(6) IADLs time 2	195	0–13	9.97	3.68	−1.06	.01
(7) Physical health time 1	199	0–3	1.95	.75	.17	−.29
(8) Physical health time 2	193	0–3	1.84	.80	.18	−.39
(9) Social resources time 1	197	0–9	7.57	1.45	−1.87	5.64
(10) Social resources time 2	190	0–9	7.40	1.55	−1.16	1.58

**Table 3:** Correlation matrix of resources (Time 1 below the diagonal; Time 2 above the diagonal).

Variables	1	2	3	4	5	6
(1) Age group	—	−.17**	−.24***	−.73***	−.10	−.29***
(2) Economic resources	−.03	—	.19**	.27***	.28***	.09
(3) Mental health	−.07	.31***	—	.20**	.34***	.10
(4) IADLs	−.73***	.15*	.15*	—	.31***	.27***
(5) Physical health	−.15*	.31***	.50***	.22**	—	.13*
(6) Social resources	−.20**	.13*	.16*	.27***	.20**	—

*P ≤ .05; **P ≤ .01; ***P ≤ .001 (one-tailed tests).

Age group scored so that 0 = 60s; 1 = 80s; 2 = 100s.

**Table 4:** Mean differences for OMFAQ resources by age group at time 1.

		*M *(SD)		*F*	*η* ^2^
Resource Construct					
	60s	80s	100s		

	(*n* = 91)	(*n* = 93)	(*n* = 133)		

Economic resources	7.86_a_	8.18_a_	7.63_a_		
	(2.29)	(1.97)	(2.25)	1.74	.001

	(*n* = 91)	(*n* = 93)	(*n* = 133)		

IADLs	12.78_a_	12.32_a_	7.81_b_		
	(.70)	(1.39)	(2.83)	214.18***	.58

	(*n* = 91)	(*n* = 93)	(*n* = 133)		

Physical health	2.02_a_	1.97_a_	1.74_b_		
	(.79)	(.71)	(.79)	4.48*	.03

	(*n* = 90)	(*n* = 93)	(*n* = 131)		

Mental health	2.16_a_	2.02_a,b_	2.02_b_		
	(.75)	(.66)	(.70)	1.23	.01

	(*n* = 91)	(*n* = 93)	(*n* = 130)		

Social resources	7.65_a_	7.94_a_	6.94_b_	13.53***	.080
	(1.23)	(.91)	(1.91)		

Means in the same row that do not share subscripts differ at *P* < .05 in the Scheffé post hoc test.

*P < .05; **P < .01; ***P < .001.

**Table 5:** Mean Changes by age group over time in OMFAQ resources.

Variable	60s		80s		100s		*F*_*T*_	*F*_T*A_	*η* ^2^
	T1	T2	T1	T2	T1	T2			
	(*n* = 69)		(*n* = 61)		(*n* = 62)				
(1) Economic resources	8.01	7.93	8.26	8.26	7.67	7.1	2.19	1.42	.01/.02
	(2.12)	(2.00)	(1.84)	(2.01)	(2.29)	(2.39)			

	(*n* = 68)		(*n* = 61)		(*n* = 58)				
(2) Mental health	2.12	2.16	2.00	1.98	1.98	1.76	1.39	2.10	.01/.02
	(.72)	(.70)	(.68)	(.72)	(.74)	(.71)			

	(*n* = 69)		(*n* = 61)		(*n* = 63)				
(3) IADLs	12.88	12.54	12.38	11.30	7.73	5.87	55.71***	9.05***	.23/.09
	(.44)	(.98)	(1.33)	(2.19)	(2.72)	(3.23)			

	(*n* = 69)		(*n* = 61)		(*n* = 62)				
(4) Physical health	2.07	1.86	2.02	1.98	1.79	1.68	4.91*	.98	.03/.01
	(.78)	(.81)	(.65)	(.67)	(.79)	(.88)			

	(*n* = 69)		(*n* = 61)		(*n* = 57)				
(5) Social resources	7.77	7.75	7.93	7.71	7.05	6.68	2.40	.62	.01/.01
	(1.19)	(1.17)	(.87)	(1.50)	(1.96)	(1.72)			

Standard deviations in parentheses.

*P < .05; ***P < .001.

**Table 6:** Univariate growth curve results for OARS resource constructs.

Outcome	Mean		Variance		
	Intercept	Slope	Intercept	Slope	*r* (intercept, slope)
(1) Economic resources	7.96***	−.23	4.46***	4.35***	−.45***
(2) Mental Health	2.03***	−.06	.51***	.58***	−.52***
(3) IADLs	12.87***	−.34***	.20*	.90***	−.17
(4) Physical health	1.96***	−.12*	.56***	.57***	−.44**
(5) Social resources	7.76***	−.01	1.38***	1.81**	−.58***

*P ≤ .05; **P ≤ .01; ***P ≤ .001.

**Table 7:** Predictors of growth factors in resources.

Time 1 predictors	Economic resources		Mental health		IADLs		Physical health		Social resources	
	Intercept	Slope	Intercept	Slope	Intercept	Slope	Intercept	Slope	Intercept	Slope
(1) Age group	.17*	−.05	.03	−.22*	−.70∗∗∗	−.31***	−.06	.12	−.02	−.32***
(2) Economic resources	—	—	.17**	−.16*	.10*	−.04	.16*	−.07	.04	.07
(3) Mental health	.20*	.01	—	—	.02	−.04	.42***	−.02	.06	−.07
(4) IADLs	. 20***	.10	.03	−.05	—	—	.06	.11	.26**	−.27*
(5) Physical health	.19*	−.08	.43***	−.14*	.04	.06	—	—	.11	−.04
(6) Social resources	.05	.05	.05	−.08	.13*	−.11	−.09	−.09	—	—

*P < .05; **P < .01; ***P < .01 (one-tailed tests).
